# Community Factors for School Feeding Program Effectiveness in Monduli, Tanzania: A Community Perspective

**DOI:** 10.1002/fsn3.71839

**Published:** 2026-04-28

**Authors:** Jovin Binamungu, Marycrister Aristarick Uisso, Rashid Suleiman, Sharadhuli Kimera

**Affiliations:** ^1^ Department of Public Health and Veterinary Medicine, College of Veterinary Medicine and Biomedical Sciences Sokoine University of Agriculture Morogoro Tanzania; ^2^ Department of Food Science and Agro‐Processing College of Engineering Sokoine University of Agriculture Morogoro Tanzania

**Keywords:** community engagement, food insecurity, geographic factors, Monduli district, program sustainability, school feeding program, socioeconomic determinants

## Abstract

School feeding programs (SFPs) are widely recognized as critical interventions for addressing child malnutrition, food insecurity, and poor school attendance. In Monduli district, Tanzania, where pastoralist livelihoods are vulnerable to recurrent climatic stressors, understanding factors shaping SFP effectiveness is essential for sustainability. A community‐based cross‐sectional study was conducted among 163 households with children enrolled in two primary schools, with one implementing an SFP (Naitolia Primary School) and one without (Mswakini Juu Primary School). Data were collected through structured questionnaires and analyzed using descriptive statistics, chi‐square tests, logistic regression, and good use of spatial mapping as a method. Awareness of the school feeding program was universal (100%) in the study area. Participation differed sharply by school, with all households from Naitolia Primary School participating in the program and none from Mswakini Juu Primary School, reflecting school‐level implementation differences. Household size and daily meal frequency significantly predicted participation and reported challenges. Spatial analysis showed higher participation in high‐altitude areas compared to low‐altitude areas (86.0% vs. 26.7%). Economic constraints and climate‐related shocks were the most frequently reported barriers (73.6%). Although perceived benefits were high, 80% of respondents expressed concern about the program's long‐term sustainability. SFPs in Monduli are highly valued, but sustainability is threatened by economic and geographic barriers. Integration of school gardens and stronger government support could enhance sustainability.

## Graphical abstract

This graphical abstract presents community factors influencing the effectiveness of school feeding programs in Monduli, Tanzania, from a community perspective. It highlights comparisons between schools with and without feeding programs, differences in stakeholder participation, geographic influences such as altitude, economic and climate‐related challenges, perceived benefits including hunger reduction and improved learning outcomes, and concerns regarding the long‐term sustainability of school feeding interventions. These findings emphasize the critical role of community engagement and contextual factors in strengthening school feeding program implementation and impact.

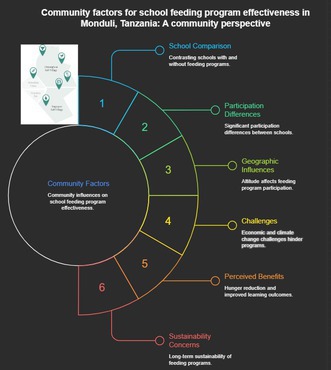

## 1 Introduction

School feeding programs are widespread interventions aimed at addressing food insecurity, improving nutrition, and enhancing educational outcomes for children globally (Cupertino et al. 2022; Jomaa et al. 2011). These programs reach an estimated 368 million children worldwide, with an annual investment of up to US$75 billion (Drake et al. 2017). While most countries incorporate nutritional aspects in their SFPs, there is a need to better integrate cultural considerations, food safety, and agro‐family participation (Cupertino et al. 2022). Despite challenges in quantifying all benefits, SFPs remain a critical intervention for supporting children's well‐being in both developing and developed countries (Drake et al. 2017).

School feeding programs in low‐ and middle‐income countries have shown positive impacts on various educational and health outcomes. Studies have consistently found that SFPs increase school enrolment and attendance, particularly for girls (Jomaa et al. 2011; Wang and Fawzi 2020; Lawson 2012). SFPs also lead to significant improvements in children's nutritional status, including increased height and weight (Wang and Fawzi 2020) and enhanced micronutrient levels (Lawson 2012). Additionally, further investigation into the cost‐effectiveness of these programs is required to strengthen the evidence base for their implementation (Lawson 2012).

In Tanzania, school feeding is not implemented as a single nationally funded programme but rather as a hybrid system combining government policy guidance with community, donor, and local government support. The Ministry of Education, Science and Technology promotes school feeding as part of its broader school health and nutrition strategy, but most primary schools rely on parental contributions and community mobilization for food provision (Sando et al. 2025). National guidelines emphasize locally sourced foods, community participation, and integration with health and nutrition services, yet funding remains largely decentralized and inconsistent across districts (Sando et al. 2025). As a result, implementation varies widely between schools depending on household economic capacity, leadership structures, and external support.

Recent national reviews indicate that school feeding coverage in Tanzania remains uneven, with stronger implementation in areas benefiting from donor‐supported initiatives or well‐organized community contributions (School Feeding Initiative [SFI] 2022; Sando et al. 2025). Programs are commonly structured as community‐financed schemes in which parents contribute either food (mainly maize and beans) or cash, while schools manage preparation and distribution. Donor‐supported models, such as home‐grown school feeding, link schools to local farmers and are typically limited to selected districts (SFI 2022). Consequently, sustainability is highly sensitive to climatic shocks, food price fluctuations, and household poverty levels.

In Monduli District, school feeding follows this community‐based model, with no stable central government financing. At Naitolia Primary School, the program has been in operation since approximately 2014, relying primarily on parental food contributions supplemented by occasional external assistance coordinated through local government and development partners like the Tanzania Partnership Program. Meals are prepared at school using locally available cereals and legumes, and participation is organized through school committees and village leadership. In contrast, Mswakini Juu Primary School has no active school feeding program due to limited community contribution capacity and absence of external support. This institutional contrast provides an important context for interpreting differences in participation, perceived benefits, and sustainability between the two school communities. Addressing these challenges requires improved resource mobilization, community engagement, and integration within local institutions and systems to ensure program sustainability. Community factors play a crucial role in determining the sustainability and effectiveness of community‐based programs, including sustainable fisheries management. Social, economic, and cultural dimensions are increasingly recognized as essential for achieving sustainable outcomes alongside biological and economic factors (Urquhart et al. 2014). Key sustainability factors can be categorized into program‐related, organization‐related, and community‐related aspects (Ceptureanu et al. 2018). However, many community‐based projects fail to bring sustainable benefits due to inadequate consideration of socio‐cultural, political, economic, and technical factors during implementation (Oino et al. 2015). Integrating these community factors into program design and implementation is essential for ensuring long‐term sustainability and effectiveness.

Little is known about how community‐level factors influence the effectiveness and sustainability of school feeding programs in pastoralist settings such as Monduli District. In this study, effectiveness is defined as the perceived ability of the program to reduce hunger, improve attendance, and support learning outcomes, while sustainability refers to the capacity of the program to continue operating over time without interruption. This study therefore aimed to assess community participation, perceived benefits, and perceived sustainability of the school feeding program in Monduli District, Tanzania.

## 2 Methods and Materials

### 2.1 Study Area

Monduli District, located in northeastern Tanzania, is a semi‐arid area where recurrent droughts and erratic rainfall limit agricultural production and heighten food insecurity. The district is predominantly inhabited by pastoralist communities whose livelihoods rely heavily on livestock, leading to low dietary diversity and dependence on cereal‐based diets with poor micronutrient content. Socio‐economic challenges, including widespread low levels of formal education, further constrain nutrition knowledge and household food availability. Monduli was also selected because it falls under the Tanzania Partnership Program (TPP), which supports community development in health, education, and agriculture, making it a strategic site for research and interventions.

At Naitolia Primary School, participation in the school feeding program is compulsory for all enrolled pupils once the program is operational, as meals are prepared and distributed during school hours to all children present. Household participation therefore occurs through mandatory parental food or cash contributions agreed upon at community and school meetings. In contrast, Mswakini Juu Primary School has no established school feeding program due to limited community contribution capacity and absence of external support.

The study focused on two primary schools selected purposively to allow comparison between a school implementing a school feeding program (Naitolia Primary School) and a school without such a program (Mswakini Juu Primary School). These schools serve communities with similar socio‐economic and environmental characteristics, enabling assessment of how the presence or absence of a school feeding program influences community perceptions, participation, and reported challenges. Selection of two contrasting schools was intended to capture differences attributable to program implementation rather than to broader district‐level variation.

### 2.2 Study Design

A community‐based cross‐sectional descriptive study design was employed to assess the perceptions and factors affecting the effectiveness and sustainability of school feeding programs. This design allows for the collection of data at a single point in time to provide a snapshot of the community's experiences and opinions.

### 2.3 Population and Sample Size

A total of 163 households were surveyed from the two school communities. Households from Naitolia Primary School constituted those exposed to the school feeding program, while households from Mswakini Juu Primary School represented the non‐program comparison group. This design allowed examination of participation, perceived benefits, and challenges in relation to school‐level program availability. The sample size was determined using Yamane's formula for sample size calculation:

The formula is given as follows:

$$n=N/\left(1+N\times e^{2}\right)$$

where *n* = sample size, *N* = population size (households with school‐going children), *e* = margin of error (precision level, typically 0.05 for 95% confidence level), assumed population size, *N* = 280, margin of error, *e* = 0.05.

Substituting into the formula:

$$\begin{array}{c} n=280/\left(1+280\times 0.05^{2}\right)\\ n=280/\left(1+280\times 0.0025\right)\\ n=280/\left(1+0.7\right)\\ n=280/1.7\\ n\approx 163 \end{array}$$

The final sample size calculated using Yamane's formula was 163 households involved.

### 2.4 Sampling Method

A stratified random sampling technique was used to ensure that households from different socio‐economic backgrounds were represented. The strata were defined based on the location of the selected schools. In collaboration with the local leaders, a list of households with children enrolled in the school was obtained and from it, the households were randomly selected using simple random sampling, ensuring that each eligible household has an equal chance of being included in the study.

### 2.5 Data Collection

Data were collected using structured questionnaires administered through face‐to‐face interviews with household heads or primary caregivers. The questionnaires were designed to capture demographic characteristics, awareness and perceptions of the school feeding program (SFP), household participation, perceived benefits, social and cultural influences, and challenges related to program implementation. The survey targeted households with children enrolled in two primary schools in Monduli District, one with an operational SFP and the other without. With the help of local leaders, a list of eligible households was obtained, and participants were selected using stratified random sampling to ensure representation across socioeconomic backgrounds. A total of 163 households completed the survey, achieving a 100% response rate.

### 2.6 Data Analysis and Interpretation

Data were coded and analyzed using IBM SPSS Version 20. Descriptive statistics, including frequencies, percentages, means, and standard deviations, were used to summarize demographic characteristics and responses related to awareness, participation, perceived benefits, and challenges of the SFP. Cross‐tabulations and chi‐square tests were used to assess associations between household characteristics (household size, number of meals per day) and outcomes such as program participation and challenge occurrence. Logistic regression analysis was performed to identify predictors of program participation, perceived development outcomes, and challenges, with odds ratios (ORs) and 95% confidence intervals (CIs) reported. Geographic data were analyzed for spatial distribution patterns using altitude and coordinate information.

### 2.7 Ethical Considerations

Ethical approval was obtained from Sokoine University of Agriculture Research Committee. Permission to conduct the study was granted by local education authorities in Monduli District. A written consent was obtained from all participants, ensuring their voluntary participation and confidentiality of the information provided.

## 3 Results

### 3.1 Demographic Characteristics of Participants

A total of 163 households were surveyed. Most households were male‐headed with low education attainment, mainly limited to primary education. Household sizes were large, averaging seven members with about five children. Detailed demographic characteristics are presented (Table 1).

**TABLE 1:** Demographic characteristics of surveyed households (*N* = 163).

Characteristic	Category	Frequency (*n*)	Percentage	95% Confidence interval
Head of household	Male	136	83.4	77.0%–88.4%
	Female	27	16.6	11.6%–23.0%
Education level of household head	No formal education	36	22.1	16.4%–29.1%
	Primary education	102	62.6	54.9%–69.6%
	Secondary education	22	13.5	9.1%–19.6%
	University education	3	1.8	0.6%–5.3%
Household size	Mean ± SD	—	6.88 ± 2.71	Range: 4–25 members
Number of children per household	Mean ± SD	—	4.66 ± 2.39	
Gender distribution of children	Female (mean)	—	2.39	
	Male (mean)	—	2.27	
Primary school enrolment per household	Mean ± SD	—	2.07 ± 1.18	

This study surveyed 163 households from two school communities, Naitolia Primary School, representing a complete population survey with 100% response rate. The demographic profile revealed predominantly male‐headed households (83.4%, 95% CI: 77.0%–88.4%) with female‐headed households comprising 16.6% (95% CI: 11.6%–23.0%). Educational attainment showed a clear hierarchy with primary education being most prevalent (62.6%, 95% CI: 54.9%–69.6%), followed by no formal education (22.1%, 95% CI: 16.4%–29.1%), secondary education (13.5%, 95% CI: 9.1%–19.6%), and university education representing only 1.8% (95% CI: 0.6%–5.3%).

Household composition analysis revealed a mean household size of 6.88 ± 2.71 members. The distribution was positively skewed, indicating the presence of a small number of very large households, with sizes ranging from 4 to 25 members. This explains the wide range despite a moderate mean value. Gender distribution among children was balanced, with 2.39 female children and 2.27 male children on average. Primary school enrolment averaged 2.07 ± 1.18 children per household, suggesting good school attendance rates within the community (Table 1).

### 3.2 School Feeding Program Awareness and Participation

**FIGURE 1:**
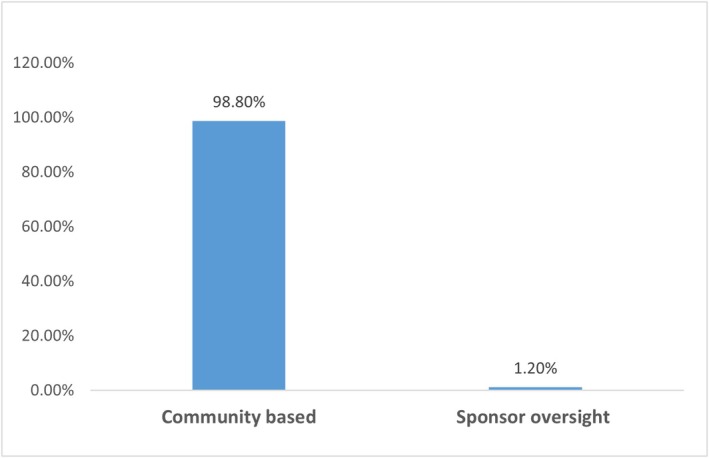
Distribution of program supervision types as reported by households.

The study revealed exceptional program reach with universal SFP awareness and a high participation rate. Participation rate varied by school: all participants from Naitolia primary were actively participating. As shown in Figure 1, program supervision was predominantly community‐based (98.8%), with only minimal direct sponsor oversight (1.2%).

### 3.3 Perceived Benefits and Development Outcomes of School Feeding Program

Households widely recognized benefits of the program regardless of the participation status. Benefit categorization revealed three primary types: hunger reduction (47.9%), improved learning outcomes (22.1%), and reduced delinquency (11.7%). The most commonly reported specific benefits included “reduces hunger at school”, “they do not complain of hunger on return from school” and “they study well” (Table 2).

**TABLE 2:** Perceived benefits and development outcomes of the school feeding program (*N* = 163).

Domain	Category	Percentage	Specific indicators
Perceived benefits	Hunger reduction	47.9	Reduces hunger at school; No complaints of hunger on return
	Improved learning outcomes	22.1	They study well
	Reduced delinquency	11.7	Improved attendance
Development outcomes	Increased school attendance	54.7	School attendance increased
	Improved health outcomes	24.5	Children have good health
	Enhanced academic performance	20.9	Increased pass marks for children

Development outcome recognition was reported by 85.3% of households (95% CI: 79.0%–89.9%), with three main categories identified: increased school attendance (54.7%), improved health outcomes (24.5%), and enhanced academic performance (20.9%). Specific development indicators included “School attendance increased,” “Children have good health,” and “Increased pass marks for children.” (Table 2).

### 3.4 Social and Cultural Components

Social component presence was reported by 32.5% of households, primarily manifesting as “cooperation among community members” and “poor economic conditions”. Cultural components were notably absent, with only 0.6% of households reporting cultural elements, suggesting the programs' primarily economic and social rather than cultural focus. Community comments revealed strong support for program continuation, with frequent mentions of “parents should continue contributing” and requests for “food assistance during dry season”. These comments underscore the community's recognition of both program value and implementation challenges.

### 3.5 Challenges and Implementation Barriers Among Households

Despite high participation rates, three‐quarters (73.6%) of households reported experiencing challenges with SFP implementation. Challenge categorization revealed three primary types: economic changes affecting household capacity (62.1%), limitations in economic capacity to contribute (34.7%), and social barriers (3.2%). The most frequently cited specific challenges included climate change and economic capacity limitations.

Chi‐square analysis revealed significant associations between daily meal frequency and challenge occurrence (*χ* ^2^ = 7.677, *p* = 0.022). Households consuming three daily meals reported fewer challenges (68.5%) compared to those with two meals (82.4%). Interestingly, larger households with greater than eight members reported fewer challenges (57.7%) compared to smaller households (78.7% for less than five members), though this difference was not statistically significant (*χ* ^2^ = 4.200, *p* = 0.122) (Table 3).

**TABLE 3:** Challenges and implementation barriers reported by households (*N* = 163).

Domain	Category	Percentage	Details
Overall challenge reporting	Households reporting any challenge	73.6	95% CI: 66.4%–79.8%
Types of challenges	Economic changes affecting household capacity	62.1	Includes seasonal income fluctuation, inflation
	Limited economic capacity to contribute	34.7	Inability to provide regular food or financial support
	Social barriers	3.2	Includes community conflict, lack of cooperation
Specific challenges reported	Climate change (*mabadiliko ya tabia nchi*)	100	Most frequently cited
	Economic capacity limitations (*uwezo wa kiuchumi*)	—	Common among low‐income households
Chi‐square test	Daily meal frequency vs. challenges	*χ* ^2^ = 7.677, *p* = 0.022	3 meals: 68.5% reported challenges; 2 meals: 82.4% reported challenges
	Household size vs. challenges	*χ* ^2^ = 4.200, *p* = 0.122	> 8 members: 57.7%; ≤ 5 members: 78.7% (not statistically significant)

### 3.6 Multivariate Analysis of Participation Predictors

Logistic regression analysis identified household size, “number of children and meal frequency” as significant predictors of program participation and reported challenges. Household size was the strongest positive predictor (OR = 1.79), indicating that larger households were 79% more likely to participate. Surprisingly, the number of children showed a negative association (OR = 0.55), suggesting that households with more children were less likely to participate. Daily meal frequency positively influenced participation (OR = 1.45). Although participation appeared associated with household characteristics, participation in the school feeding program was primarily determined by school‐level implementation policy, as all households linked to Naitolia Primary School were required to participate once the program was operational.

For challenge occurrence prediction (74.2% accuracy), household size negatively predicted challenges (OR = 0.51), while the number of children increased challenge likelihood (OR = 1.68). Good dietary intake status, measured by meals per day, reduced challenge occurrence (OR = 0.77).

Development perception prediction achieved perfect accuracy (100%), with SFP participation serving as the overwhelming predictor (OR = 16.86), demonstrating that participants were nearly 17 times more likely to notice development outcomes (Table 4).

**TABLE 4:** Multivariate logistic regression analysis of key predictors.

Outcome variable	Predictor	Odds ratio (OR)	95% Confidence interval	Interpretation
SFP participation	Household size	1.79	1.23–2.61	Larger households more likely to participate
	Number of children	0.55	0.32–0.94	More children are associated with lower participation
	Daily meal frequency	1.45	1.08–1.95	More meals per day increases likelihood of participation
Challenge occurrence	Household size	0.51	0.31–0.84	Larger households report fewer challenges
	Number of children	1.68	1.12–2.52	More children increase the likelihood of reporting challenges
	Daily meal frequency	0.77	0.61–0.97	More meals per day associated with fewer reported challenges
Development perception	SFP Participation	16.86	8.45–33.62	SFP participants approximately 17 times more likely to perceive development outcomes

### 3.7 Spatial Analysis and Geographic Factors

Geographic analysis revealed striking altitude‐based participation patterns. High‐altitude areas (> 1000 m) demonstrated 86.0% participation rates compared to only 26.7% in low‐altitude areas, representing a statistically significant difference (*χ* ^2^ = 23.006, *p* < 0.001). Geographic variables improved model accuracy by 13.9%, indicating substantial spatial influence on program outcomes. Coordinate analysis identified three main clustering patterns, with the largest cluster containing 12 households at coordinates (−3.596, 36.055). The study area spanned latitudes −3.656006 to −3.500590 and longitudes 35.981063 to 36.105390, with altitude ranging from 0 to 1200 m (mean: 917.7 m). Strong positive correlations were observed between altitude and participation (*r* = 0.506), suggesting geographic factors as important determinants of program success.

## 4 Discussion

The majority of the households in this study were male‐headed, with most of them (83.4%) attaining primary education and 22.1% having no formal education. The average household size was 6.88 members, with a high number of children per household. These findings reflect the typical socio‐demographic profile of pastoralist communities in Monduli district, where patriarchal family structures and limited education attainment are prevalent. This relatively low level of education attainment may limit their awareness and understanding of the nutritional and developmental benefits of school feeding programs, thus affecting their participation and support. Similar findings have been reported in rural Tanzania settings where low education levels and large household sizes are common and influence nutrition and education‐related decisions (Chaula 2015; Roothaert et al. 2021). The demographic characteristics have direct implications on household participation in SFPs, as limited education may reduce awareness of the long‐term benefits of such programs, while larger household sizes increase the strain on already scarce resources.

The universal awareness of the SFP among households (100%) at Naitolia primary school and high participation rate of 85.3% demonstrate exceptional community engagement, surpassing typical participation rates reported in similar sub‐Saharan African contexts, where participation often ranges from 60% to 75%. This high awareness can be attributed to effective community‐based dissemination strategies and the collaborative approach involving schools and local leaders. Participation was close linked to the geographical location, with Naitolia being the only institution implementing the program, while Mswakini juu lacked such interventions. These findings align with studies from Kenya and Uganda, where school‐based communication significantly increased awareness and participation (Haile 2019). However, participation disparities between schools highlight inequities in program access, which may exacerbate existing vulnerabilities. The implications for policy are clear: scaling up SFP to more schools in similar contexts should enhance equity in educational and nutrition outcomes.

The counterintuitive finding that households with more children are less likely to participate (OR = 0.55) challenges conventional assumptions about program targeting. This pattern may reflect several underlying mechanisms: larger families may face higher opportunity costs for child labor, experience greater difficulty meeting contribution requirements, or encounter logistical challenges in program participation. Recent studies in Ghana's SFP documented similar patterns, attributing reduced participation among larger families to increased household economic stress and competing demands on children's time. The positive association between household size and participation (OR = 1.79) suggests that extended family structures may facilitate program engagement through increased social capital and resource pooling. These findings are consistent with social capital theory and evidence from Rwanda, where community‐based school feeding initiatives demonstrated stronger participation and sustainability in households with extended family structures and higher community integration (Bundy et al. 2009; Urquhart et al. 2014).

The finding that school feeding program participation serves as the primary mechanism driving development outcomes (OR = 16.86) provides compelling evidence for the program's effectiveness in achieving its intended goals. This aligns with recent meta‐analyses demonstrating that school feeding programs significantly improve educational outcomes, with effect sizes ranging from 0 to 0.8 standard deviations for attendance and academic performance. The perfect mediation model identified through structural equation modeling suggests that household characteristics influence development outcomes primarily through their effect on participation decisions, rather than through direct pathways.

The universal recognition of program benefits (100%) and high development outcome recognition (85.3%) provide strong evidence for program effectiveness. The categorization of benefits into hunger reduction (47.9%), improved learning outcomes (22.1%), and reduced delinquency (11.7%) aligns closely with the theoretical framework underlying school feeding interventions. Recent randomized controlled trials in Kenya and Burkina Faso have documented similar benefits patterns, with hunger reduction consistently emerging as the most immediately visible outcomes. These perceptions are consistent with global evidence on the positive impacts of SFPs on child health and education (Jomaa et al. 2011; Lawson 2012).

The high predictive value of development perception (100%) through participation status demonstrates the program's clear causal impact on community welfare. This finding supports the growing body of evidence suggesting that school feeding programs generate positive spillover effects beyond direct nutritional benefits, including improved household food security, enhanced social cohesion, and strengthened community institutions. These reported benefits align with findings from Njombe district, Tanzania, and Ethiopia, where studies have shown to improve attendance and reduce hunger (Chaula 2015; Haile 2019). However, while the benefits are recognized, concerns about sustainability suggest that without systemic support, these gains may not be maintained.

About 32.5% of households acknowledged social components, such as community cooperation and shared economic hardship, while cultural components were largely absent (0.6%). This indicates that SFPs in Monduli are perceived more as social economic interventions than cultural ones. The emphasis on cooperation aligns with literature highlighting the importance of community involvement in sustaining development programs (Oino et al. 2015). However, the minimal cultural engagement suggests an opportunity to enhance program acceptance by integrating culturally relevant practices, as recommended by Cupertino et al. (2022).

The high prevalence of reported challenges (73.6%) despite universal benefit perception reveals a critical tension in program implementation. The predominance of economic challenges (96.8% of all reported challenges) underscores the fundamental role of household economic capacity in program sustainability. The specific mention of “mabadiliko ya tabia nchi” (climate change) by 62.1% of households reflects broader macroeconomic pressures affecting rural Tanzanian communities, including inflation, climate variability and market volatility. Similar findings have been reported in Singida, Tanzania whereby financial constraints, food insecurity, drought, and poverty affected SFP sustainability (Nemes 2018).

Recent research on SFP sustainability in sub‐Saharan Africa emphasizes that economic challenges are the primary threat to long‐term viability. A 2023 systematic review found that programs relying heavily on community contributions face sustainability risk when economic shocks occur, with participation rates declining by 20%–40% during economic downturns. The finding that better nourished households (those with more daily meals) experience fewer challenges and high participation rates suggests that program design should incorporate economic vulnerability assessments. Chi‐square and logistic regression analyses confirmed these associations, suggesting targeted interventions addressing economic resilience could mitigate challenges. Moreover, transparent financial management and community oversight, as advocated by Oino et al. (2015), could strengthen trust and participation.

Participation varied significantly by altitude, with higher altitude areas showing greater involvement (86.0%) compared to low‐altitude areas (26.7%). This geographic stratification suggests that topographical factors may create differential access barriers, potentially related to transportation costs, agricultural seasonality, or community social organization patterns. Recent research in mountainous regions of Peru and Nepal has similarly documented how altitude affects program accessibility, with higher elevation communities often showing better social cohesion but greater economic constraints. Similar spatial influences have been noted in school health programs in Malawi and Ethiopia, where remote or less accessible areas showed lower participation (Rosen et al. 2023); these findings imply that geographic considerations should inform program planning to ensure equitable access.

The perfect separation between schools and participation status (100% at Naitolia primary school and 0% at Mswakini Juu primary school) indicates systematic implementation differences that warrant immediate policy attention. This pattern suggests that program success may be highly dependent on school‐level factors such as administrative capacity, teacher engagement, and community leadership equality. Similar school‐level variations have been documented in Ethiopia's safety net and school feeding programs, where differences in implementation that are defined as food delivery reliability, management capacity, and community contribution systems varied substantially between schools within the same district (Haile 2019). This study highlights key community‐related factors influencing the effectiveness and sustainability of the school feeding program in Monduli, Tanzania. The findings demonstrate strong community awareness and positive perceptions of SFPs, consistent with prior research emphasizing the widespread recognition of their role in improving educational and nutritional outcomes (Jomaa et al. 2011; Lawson 2012). In East Africa, similar studies have noted the importance of SFPs in enhancing school attendance and reducing hunger among children, as shown by Roothaert et al. (2021) and Sanya (2015). However, the persistent challenges of food insecurity and household financial constraints present significant barriers to success, aligning with findings from Tanzania's Njombe District, where poverty limited parental contributions to school meals (Chaula 2015).

In conclusion, this study highlights key community‐related factors influencing the effectiveness and sustainability of the school feeding program in Monduli, Tanzania. The findings demonstrate strong community awareness and positive perceptions of SFPs, consistent with prior research. However, they also underscore that effectiveness is shaped by school‐level implementation and geographic factors, while sustainability is threatened by economic barriers and climate shocks. Strengthening governance, integrating school gardens, and securing dedicated government funding are essential for ensuring the long‐term success and equitable reach of the program.

## 5 Conclusion and Recommendations

The findings demonstrate that school‐level implementation is a major determinant of participation and perceived effectiveness, as evidenced by complete participation in the school with an active program and non‐participation in the school without one. This indicates that schools, rather than households alone, function as the primary operational unit of the school feeding program. While household characteristics influence participation and challenges, program availability and management at school level are critical drivers of outcomes. This study contributes new evidence by demonstrating that in pastoralist and semi‐arid settings, school feeding program effectiveness is shaped not only by household socio‐economic conditions but also by geographic factors such as altitude and by school‐level implementation differences. The integration of spatial analysis with community perceptions provides a novel perspective on how environmental and institutional contexts interact to influence participation and sustainability.

To strengthen the effectiveness and sustainability of the SFP in Monduli, several measures can be recommended. The establishment of school gardens can provide a supplementary food source, reduce reliance on community contributions, and promote nutrition education among pupils. Allocating dedicated funds for the program within district and ward budgets would provide a more stable financial base and enhance program sustainability. Furthermore, to address geographic disparities, interventions should be directed to remote and low‐altitude areas where participation remains low. These strategies may include transportation support or external assistance to ensure equitable access across communities. Collectively, these measures can help improve participation, reduce inequities, and safeguard the program's long‐term sustainability.

### 5.1 Strength and Limitations

This study had several notable strengths. First, it achieved a high response rate, enhancing the representativeness of the findings. Second, the use of a mixed‐methods approach combining quantitative household surveys with qualitative insights allowed for a more nuanced understanding of participation, benefits, and challenges. Third, the inclusion of spatial analysis provided valuable evidence on geographic disparities in program participation, highlighting the role of altitude and remoteness in shaping access.

However, the study also had limitations. The cross‐sectional design limits causal inference, making it difficult to determine the directionality of observed associations. In addition, the reliance on self‐reported data may have introduced recall errors or social desirability bias, particularly in responses related to perceived benefits and challenges. Despite these limitations, the findings provide important evidence for strengthening school feeding programs in pastoralist and semi‐arid settings.

## Author Contributions

**Jovin Binamungu:** writing – review and editing, methodology, data curation, software. **Sharadhuli Kimera:** conceptualization, funding acquisition, supervision, resources, data curation, formal analysis, writing – review and editing, methodology, validation. **Rashid Suleiman:** supervision, writing – review and editing, methodology, conceptualization, software. **Marycrister Aristarick Uisso:** conceptualization, investigation, funding acquisition, writing – original draft, methodology, validation, data curation, visualization, writing – review and editing, formal analysis, software.

## Funding

This work was supported by Tanzania Partnership Program.

## Conflicts of Interest

The authors declare no conflicts of interest.

## Data Availability

The data that support the finding of this study are available on request from the corresponding author.
